# Oxidation of DJ-1 Induced by 6-Hydroxydopamine Decreasing Intracellular Glutathione

**DOI:** 10.1371/journal.pone.0027883

**Published:** 2011-11-21

**Authors:** Akiko Miyama, Yoshiro Saito, Kazunori Yamanaka, Kojiro Hayashi, Takao Hamakubo, Noriko Noguchi

**Affiliations:** 1 Department of Medical Life Systems, Faculty of Medical and Life Sciences, Doshisha University, Tatara, Kyotanabe, Kyoto, Japan; 2 Laboratory of Systems Biology and Medicine, Research Center for Advanced Science and Technology, University of Tokyo, Meguro-ku, Tokyo, Japan; Mental Health Research Institute of Victoria, Australia

## Abstract

DJ-1, the causative gene of a familial form of Parkinson's disease (PD), has been reported to undergo preferential oxidation of the cysteine residue at position 106 (Cys-106) under oxidative stress; however, details of the molecular mechanisms are not well known. In the present study, mechanisms of DJ-1 oxidation induced by 6-hydroxydopamine (6-OHDA) were investigated by using SH-SY5Y cells. The treatment of these cells with 6-OHDA caused an obvious acidic spot sift of DJ-1 due to its oxidation. However, when catalase, which is an hydrogen peroxide (H_2_O_2_)-removing enzyme, was added during the treatment, it failed to prevent the oxidation induced by 6-OHDA, suggesting that electrophilic *p*-quinone formed from 6-OHDA, but not H_2_O_2_, was responsible for the DJ-1 oxidation. Benzoquinone, another electrophilic *p*-quinone, also induced DJ-1 oxidation. The intracellular glutathione (GSH) levels were significantly decreased by 6-OHDA, irrespective of the presence or absence of catalase. The inhibition of GSH synthesis by buthionine sulfoximine resulted in a decrease in GSH levels and enhancement of DJ-1 oxidation. The pretreatment of cells with N-acetyl-cysteine prevented the loss of intracellular GSH and subsequently DJ-1 oxidation induced by 6-OHDA. Collectively, these results suggest that electrophilic *p*-quinone formed from 6-OHDA induces DJ-1 oxidation by decreasing intracellular GSH.

## Introduction

Parkinson's disease (PD) is a progressive, age-related, neurodegenerative disorder that is characterized by bradykinesia, rigidity, tremors, and gait dysfunction with postural instability [1]. The pathological hallmark of PD is the degeneration of dopamine neurons in the substantia nigra pars compacta and the subsequent depletion of striatal dopamine [2]. Autopsy studies have revealed that a pathological sign of PD is the presence of insoluble clumps of protein, called Lewy bodies, and a characteristic pattern of Lewy bodies in PD brain has been suggested [3]. Although the etiology of PD remains unknown, increasing evidence suggests that oxidative stress is an important mediator in its pathogenesis [4]. Oxidative stress is defined as an imbalance between oxidants and antioxidants in favor of the oxidants, potentially leading to damage [5]. It is thought that nigral dopaminergic neurons are rich in reactive oxygen species (ROS) because both enzymatic and non-enzymatic metabolism of dopamine itself leads to the generation of ROS including superoxide anion, hydrogen peroxide (H_2_O_2_), and hydroxyl radicals [6]. Indeed, there are several observations, such as the increased levels of the oxidation products of lipids, proteins, and nuclear acids in nigral cells, that are indicative of the role of oxidative stress in PD [4], [6], [7]. Additionally, it has also been known that a decrease in antioxidant defense such as cellular glutathione (GSH) is observed in the nigral lesion of PD [8].

Recent studies, particularly in the field of genetics, have identified mutations causing a familial form of PD. The DJ-1 gene has been implicated as one of the causative genes in a familial form of PD, namely, *PARK7* [9]. Mutations in *PARK7* can cause autosomal recessive parkinsonism, and the clinical presentation of the early onset and slow progression of this form of parkinsonism is similar to that of the other recessive PD syndromes such as *PARK2* (parkin) and *PARK6* (PTEN-induced kinase 1, PINK1) [9]. DJ-1 is a multifunctional protein involved in several processes such as transcriptional regulation and antioxidative defense [10], [11], [12]. Recently, the cytoprotective role of DJ-1 in dopaminergic neurons has been demonstrated [13].

Previous studies have revealed that the Cys residue at position 106, i.e., Cys-106, is oxidized to cysteine sulphonic acid (Cys-SO_3_H) in cells exposed to oxidative stress [14]. Cysteine forms 3 different species, namely, cysteine-sulfenic acid (Cys-SOH), cysteine-sulfinic acid (Cys-SO_2_H), or cysteine-sulfonic acid (Cys-SO_3_H) through direct oxygen addition. 2D-PAGE has shown the acidic spot shift of DJ-1 for cells under oxidative stress, and previous studies have shown that these acidic pI shifts are due to a post-translational process induced by the oxidation of the cysteine residue to Cys-SO_2_H or Cys-SO_3_H [14], [15]. We have developed specific antibodies against Cys-106-oxidized DJ-1 (oxDJ-1) [16]. By using a competitive enzyme-linked immunosorbent assay (ELISA) for detecting oxDJ-1, we found that the levels of oxDJ-1 in the erythrocytes of unmedicated PD patients were markedly higher than those in the erythrocytes of medicated PD patients and healthy subjects [16]. Furthermore, we recently demonstrated that animal models of PD prepared by administration of neurotoxins such as 6-hydroxydopamine (6-OHDA) and 1-methyl-4-phenyl-1,2,3,6-tetrahydropyridine (MPTP) involved the oxidative modification of DJ-1 in the brain and erythrocytes [17]. However, the molecular mechanism through which DJ-1 is oxidized is still unclear.

In order to elucidate the molecular pathways of neuronal cell death and to develop neuroprotective strategies, a number of *in vitro* and *in vivo* PD models have been characterized. 6-OHDA is a selective catecholaminergic neurotoxin that has been widely used to produce PD models *in vitro* and *in vivo*, and it is known to induce a toxicity that mimics the neuropathological and biochemical characteristics of PD [18]. It has been reported that 6-OHDA is oxidized rapidly by molecular oxygen to generate the superoxide anion, hydrogen peroxide, and 2-hydroxy-5-(2-aminoethyl)-1,4-benzoquinone (*p*-quinone) as follows [19]:
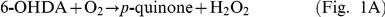
It is thought that the ROS and *p*-quinone mediates 6-OHDA-induced cell death [20]. It has been known that 6-OHDA is readily oxidized within a few minutes to produce H_2_O_2_ and *p*-quinone in the extracellular fluid rather than in the intracellular fluid [19], [20], [21]. Since it has been shown that catalase, which is barely incorporated into cells, completely removes the cytotoxic effects of H_2_O_2_, it is considered that the cytotoxicity of 6-OHDA in the presence of catalase might be primarily mediated by *p*-quinone [20], [21]. It has been shown that H_2_O_2_ generated by 6-OHDA plays a pivotal role in 6-OHDA-induced peroxiredoxin oxidation and cytochrome *c* release, while H_2_O_2_- and cytochrome *c*-independent caspase activation pathways are also involved in 6-OHDA-induced neurotoxicity [21]. It is believed that the latter cytotoxic activity, which is estimated from the cytotoxicity of 6-OHDA in the presence of catalase, is mediated by *p*-quinone.[Fig pone-0027883-g001]

**Figure 1 pone-0027883-g001:**
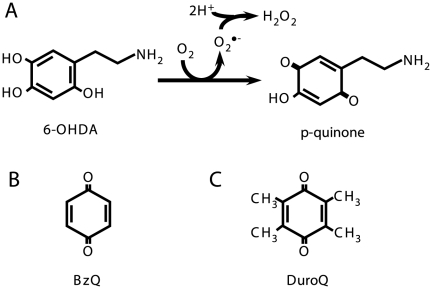
6-OHDA and quinones used in this study.

Quinones are biologically active compounds and all quinones are redox cycling agents that generate ROS. In contrast, partially substituted quinones including *p*-quinone can function as arylating agents that react with cellular nucleophiles such as thiols, thereby forming covalently linked quinone-thiol Michael adducts [22]. Arylating quinones have unique biological properties such as high cytotoxicity that are not commonly shared by non-arylating quinones and arylated thiol adducts. It has been shown that GSH is capable of reacting with *p*-quinone at the second-position to form 2-S-(glutathionyl)-6-OHDA [23]. It has also been reported that the GSH and N-acetyl cysteine (NAC) effectively attenuate the 6-OHDA-induced cytotoxicity in cultured cells [21], [24], [25].

In the present study, by using SH-SY5Y neuroblastoma cells, we investigated the mechanisms of DJ-1 oxidation induced by 6-OHDA, particularly focusing on the role of H_2_O_2_ and *p*-quinone generated by 6-OHDA. We found that electrophilic *p*-quinone, but not H_2_O_2_, plays a significant role in DJ-1 oxidation through a decrease in cellular GSH.

## Results

### Western blot analysis of hydrogen peroxide-induced DJ-1 oxidation

**Figure 2:**
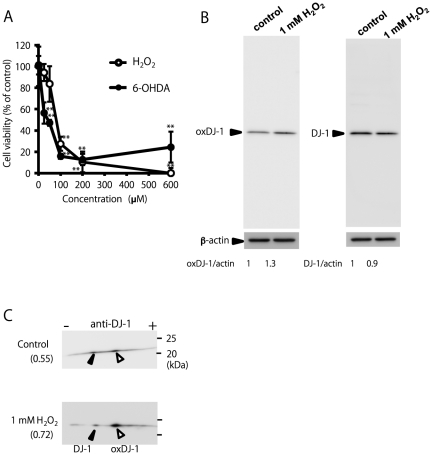
Cytotoxicity and DJ-1 oxidation induced by hydrogen peroxide. (A) SH-SY5Y cells were treated with H_2_O_2_ or 6-OHDA at various concentrations for 20 h and subjected to the MTT assay. ** Significantly different from the value of 0 µM (n = 3, *p*<0.01, Tukey, ANOVA). (B) Cell samples (15 µg) obtained from the cells treated with 0 or 1 mM H_2_O_2_ for 30 min were separated by 1D-PAGE and subsequently subjected to western blot analysis using antibodies against oxidized DJ-1, DJ-1, and βeta-actin. The number under each condition indicates the ratio of oxidized DJ-1 or DJ-1 to βeta-actin calculated from intensity (n = 2). (C) Cell samples (15 µg) obtained from the cells treated with 0 or 1 mM H_2_O_2_ for 30 min were separated by 2D-PAGE and subsequently subjected to western blot analysis using antibody against DJ-1. The filled triangle and open triangle indicate native and oxidized DJ-1, respectively. The number under each condition indicates the ratio of oxidized DJ-1 calculated from the following equation: the ratio = (intensity of oxidized DJ-1)/(intensity of DJ-1 + intensity of oxidized DJ-1) (n = 2).

To investigate the mechanism of DJ-1 oxidation induced by 6-OHDA, we first measured the effects of 6-OHDA and H_2_O_2_, which is produced by autooxidation of 6-OHDA, on the viability of human neuroblastoma SH-SY5Y cells. 6-OHDA exhibited significant cell death at concentrations higher than 25 µM, while H_2_O_2_ showed cytotoxicity at concentrations higher than 100 µM (Fig. 2A). It has been known that a relatively high concentration of H_2_O_2_ can induce DJ-1 oxidation in some types of cells, including SH-SY5Y, HUVEC, and Jurkat cells [10], [14], [26]. Therefore, SH-SY5Y cells were treated with 1 mM H_2_O_2_ for 30 min, and the cell lysates were then subjected to western blot analysis using a monoclonal antibody specific to oxDJ-1. As a result, a slight but significant increase in the immunoreactivity to the anti-oxDJ-1 antibody was observed (Fig. 2B). On the basis of the intensities, the levels of beta-actin were same (Fig. 2B), while the ratio of the levels of oxidized DJ-1 per total DJ-1 in the H_2_O_2_-treated cells to that in the control cells was calculated to be 1.45 (Fig. 2B). Same samples were separated by 2D-PAGE and subjected to western blot analysis using anti-DJ-1 antibody. We found an obvious acidic spot shift of DJ-1 in H_2_O_2_-treated SH-SY5Y cells, and the ratio of the amount of oxDJ-1 to the total amount of DJ-1 increased from 0.55 to 0.72 (Fig. 2C). On the basis of these results, we further examined DJ-1 oxidation by using a combination of western blot analysis and 2D-PAGE.

### DJ-1 oxidation induced by 6-OHDA in SH-SY5Y cells and primary cortical neuronal cells

**Figure 3:**
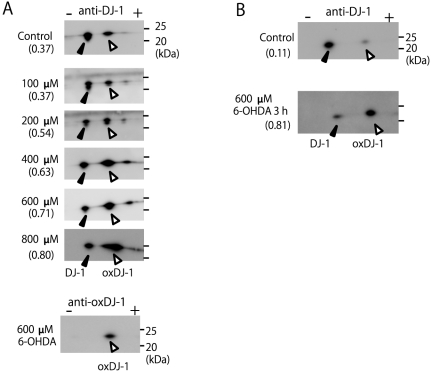
DJ-1 oxidation induced by 6-OHDA. (A) Cell samples obtained from the SH-SY5Y cells treated with 6-OHDA at indicated concentrations for 3 h were separated by 2D-PAGE and subsequently subjected to western blot analysis using antibodies against DJ-1 and oxidized DJ-1. (B) Cell samples obtained from the primary neuronal cells treated with 600 µM 6-OHDA for 3 h were separated by 2D-PAGE and subsequently subjected to western blot analysis using the anti-DJ-1 antibody. The ratio of oxidized DJ-1 is shown under each condition (n = 2).

SH-SY5Y cells were treated with variable concentrations of 6-OHDA for 3 h, and the cell lysates were then subjected to a combination of western blot analysis and 2D-PAGE. The acidic spot sift of DJ-1 increased in a concentration-dependent manner and the ratio of the amount of oxDJ-1 to the total amount of DJ-1 increased from 0.37 to 0.80 (Fig. 3A). The oxidation of DJ-1 at the acidic spot position was confirmed by using the anti-oxDJ-1 antibody (Fig. 3A). It was also confirmed that the oxidation of DJ-1 was induced when primary cortical neuronal cells were treated with 600 µM 6-OHDA for 3 h (Fig. 3B).

### Possible role of electrophilic *p*-quinone in DJ-1 oxidation induced by 6-OHDA

**Figure 4:**
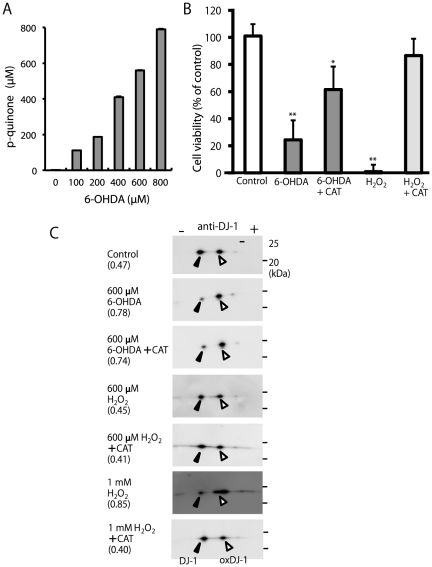
Possible role of electrophilic *p*-quinone in DJ-1 oxidation induced by 6-OHDA. (A) The determination of *p*-quinone from 6-OHDA. The formation of *p*-quinone was monitored at 490 nm in the serum medium (n = 3). (B) Cells were treated with 1 mM H_2_O_2_ or 600 µM 6-OHDA in the presence or absence of 50 U/ml catalase (CAT) for 20 h and subjected to the MTT assay. *, ** Significantly different from the value of control (n = 3, * *p*<0.05, ** *p*<0.01, Tukey, ANOVA). (C) Cell samples obtained from the SH-SY5Y cells treated under indicated conditions for 3 h were separated by 2D-PAGE and subsequently subjected to western blot analysis using the anti-DJ-1 antibody. The ratio of oxidized DJ-1 is shown under each condition (n = 2).

It has been reported that 6-OHDA is readily oxidized in the presence of oxygen to yeild H_2_O_2_ and *p*-quinone [19]. To determine the role of autooxidation in 6-OHDA-induced DJ-1 oxidation, we first determined the formation of H_2_O_2_ and *p*-quinone from 6-OHDA in cultured medium without cells. Using a molecular extinction coefficient of 1892 M^−1^ cm^−1^ at 490 nm for *p*-quinone [27], the formation of *p*-quinone from 6-OHDA was determined spectrophotometrically. This reaction was completed within 10 min. The results confirmed that *p*-quinone is formed from 6-OHDA in a concentration-dependent manner (Fig. 4A). Formation of H_2_O_2_ in cultured medium was quantified on the basis of ferrous oxidation of xylenol orange (FOX). Formation of 260 µM H_2_O_2_ from 600 µM 6-OHDA was detected, and complete disappearance of H_2_O_2_ following the addition of catalase was confirmed (data not shown).

To determine the ROS responsible for cytotoxicity induced by 6-OHDA, the effects of catalase, H_2_O_2_-removing enzyme, were examined. In the presence of 50 U/ml catalase, 1 mM H_2_O_2_ did not induce cell death (Fig. 4B). In the case of 6-OHDA, a significant but partial protective effect of catalase was observed (Fig. 4B). This result suggested the involvement of H_2_O_2_-independent cytotoxicity, which might be mediated by 6-OHDA-derived *p*-quinone.

To identify the ROS responsible for the DJ-1 oxidation induced by 6-OHDA, total lysates of the cells treated with 6-OHDA in the presence or absence of catalase for 3 h were subjected to a combination of western blot analysis and 2D-PAGE for the determination of the DJ-1 oxidation status. As shown in Fig. 4C, no obvious effect was observed in the cells treated with 600 µM H_2_O_2_, whose concentration is equal to the stoichiometric value of H_2_O_2_ formed from 600 µM 6-OHDA via autoxidation (Fig. 1A); on the other hand, 1 mM H_2_O_2_ treatment resulted in a significant increase in oxDJ-1 levels. Further, catalase treatment failed to inhibit the DJ-1 oxidation induced by 6-OHDA (Fig. 4C).

To elucidate the role of other ROS such as superoxide anion and nitric oxide on DJ-1 oxidation, we examined additional effects of superoxide dismutase (SOD) and L-N^G^-monomethyl arginine (L-NMMA), inhibitor of nitric oxide synthetase. We found that SOD and L-NMMA were not effective in preventing DJ-1 oxidation induced by 6-OHDA (Supplemental Fig. S1A and S1B), which suggested a less prominent role of superoxide anion and nitric oxide in DJ-1 oxidation induced by 6-OHDA. Collectively, these observations suggest that electrophilic *p*-quinone is responsible for the DJ-1 oxidation induced by 6-OHDA.

**Figure 5:**
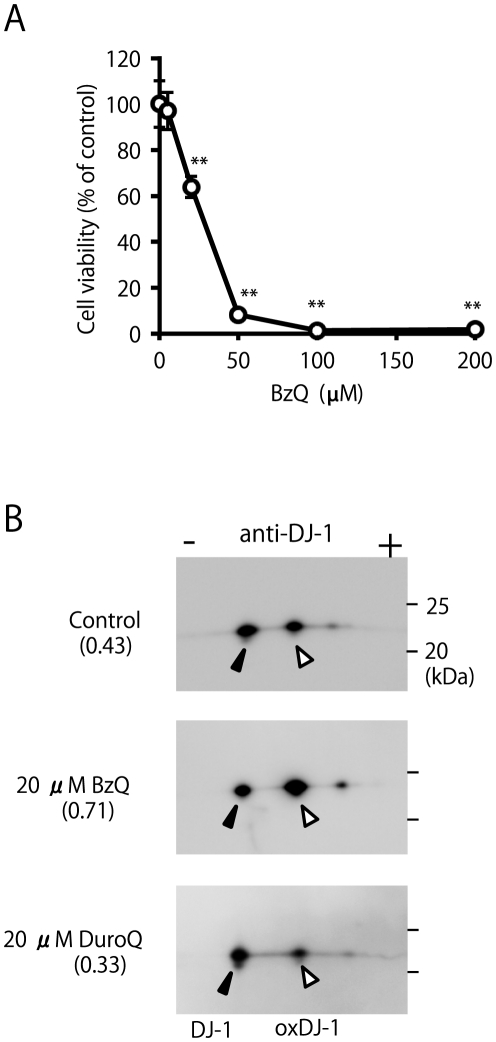
Cytotoxicity and DJ-1 oxidation induced by electrophilic benzoquinone (BzQ). (A) Cells were treated with BzQ at various concentrations for 20 h and subjected to the MTT assay. ** Significantly different from the value of 0 µM (n = 3, *p*<0.01, Tukey, ANOVA). (B) Cell samples obtained from the SH-SY5Y cells treated with the indicated conditions for 3 h were separated by 2D-PAGE and subsequently subjected to western blot analysis using the anti-DJ-1 antibody. The ratio of oxidized DJ-1 is shown under each condition (n = 2).

To investigate the importance of electrophilic properties in DJ-1 oxidation, we examined the effects of electrophilic benzoquinone (BzQ, Fig. 1B) and saturated, non-electrophilic tetramethylquinone (DuroQ, Fig. 1C) on the DJ-1 oxidation status. It has been known that electrophilic quinone has unique biological properties such as high cytotoxicity [28], [29]. In accordance with previous reports, BzQ showed significant cell death at concentrations higher than 20 µM (Fig. 5A). Total lysates of the cells treated with 20 µM BzQ or DuroQ for 3 h were subjected to the analysis of DJ-1 oxidation by using a combination of western blot analysis and 2D-PAGE. It was found that electrophilic BzQ induced DJ-1 oxidation, while non-electrophilic DuroQ did not (Fig. 5B). This observation suggests the importance of the electrophiles in 6-OHDA-induced DJ-1 oxidation.

### Significant decrease in cellular GSH induced by 6-OHDA and its role in DJ-1 oxidation

**Figure 6:**
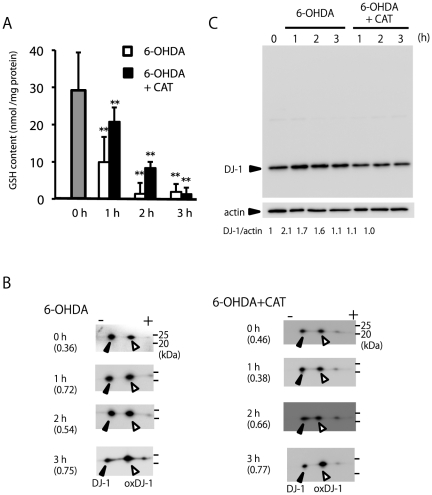
Decrease in GSH levels and DJ-1 oxidation induced by 6-OHDA with or without catalase. (A) Cells were treated with 600 µM 6-OHDA in the presence or absence of 50 U/ml catalase (CAT) for the indicated times and subjected to the GSH assay. ** Significantly different from the value at time 0 (n = 3, *p*<0.01, Tukey, ANOVA). (B) Cell samples treated under the indicated conditions were separated by 2D-PAGE and subsequently subjected to western blot analysis using the anti-DJ-1 antibody. The ratio of oxidized DJ-1 is shown under each condition (n = 2). (C) Cell samples treated under the indicated conditions were separated by 1D-PAGE and subsequently subjected to western blot analysis using antibodies against DJ-1 and βeta-actin. The number under each condition indicates the ratio of DJ-1 to βeta-actin calculated from intensity (n = 2).

Electrophilic quinone can function as an arylating agent and reacts with cellular nucleophiles such as thiols to form covalently linked quinone-thiol Michael adducts [22], [28]. Cellular GSH is one of the major nucleophiles. We next determined the change in cellular GSH levels resulting from the stimulus-inducing DJ-1 oxidation. 6-OHDA treatment resulted in a decrease in cellular GSH in a time-dependent manner, irrespective of the presence of catalase (Fig. 6A). In both cases, an increase in the oxDJ-1 ratio was observed (Fig. 6B). Slight but siginificantly elevated total DJ-1 levels were observed in 6-OHDA-treated cells, while the expression level of total DJ-1 did not change after treatment with 6-OHDA and catalase (Fig. 6C). It is notable that treatment of 6-OHDA with catalase for 1 h resulted in a significant decrease in cellular GSH but not DJ-1 oxidation. This observation suggests that a decrease in cellular GSH precedes before DJ-1 oxidation.

**Figure 7:**
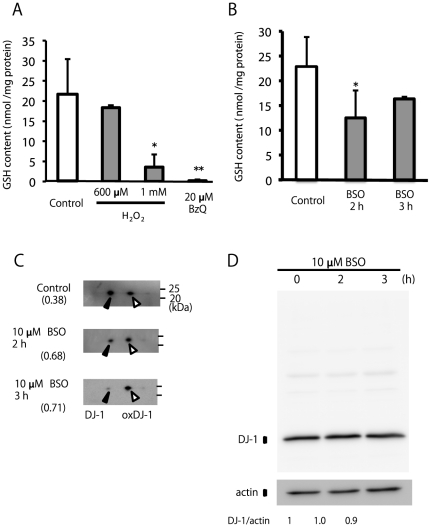
Effects of H_2_O_2_, BzQ, and BSO on the DJ-1 oxidation status. (A) SH-SY5Y cells were treated with 600 µM, 1 mM H_2_O_2_, and 20 µM BzQ for 3 h and subjected to the GSH assay. ** Significantly different from the value at time 0 (n = 3, *p*<0.01, Tukey, ANOVA). (B) Cells were treated with 10 µM BSO for indicated times and subjected to the GSH assay. ** Significantly different from the value at time 0 (n = 3, *p*<0.01, Tukey, ANOVA). (C) Cell samples obtained from SH-SY5Y cells treated with 10 µM BSO for indicated times were separated by 2D-PAGE and subsequently subjected to western blot analysis using the anti-DJ-1 antibody. The ratio of oxidized DJ-1 is shown under each condition (n = 2). (D) Cell samples treated under the indicated conditions were separated by 1D-PAGE and subsequently subjected to western blot analysis using antibodies against DJ-1 and βeta-actin. The number under each condition indicates the ratio of DJ-1 to βeta-actin calculated from intensity (n = 2).

Cellular GSH content was also determined in cells treated with the other stimuli, including H_2_O_2_ and BzQ. A significant decrease in cellular GSH levels was observed in 1 mM H_2_O_2_- and 20 µM BzQ-treated cells, but not in 600 µM H_2_O_2_-treated cells (Fig. 7A). We further examined the effect of BSO, an inhibitor of the rate-limiting enzyme of cellular GSH synthesis. Treatment with 10 µM BSO resulted in a significant decrease in cellular GSH levels (Fig. 7B) and the enhancement of DJ-1 oxidation (Fig. 7C). The expression level of total DJ-1 during treatment with BSO did not change (Fig. 7D). This result suggests that a decrease in cellular GSH can increase DJ-1 oxidation.

### Protective effects of NAC against DJ-1 oxidation induced by 6-OHDA

**Figure 8:**
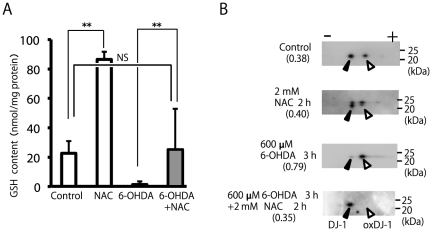
Protective effects of NAC against DJ-1 oxidation induced by 6-OHDA. (A) Cells were pretreated with or without 2 mM NAC for 2 h, and this was followed by treatment with 600 µM 6-OHDA for an additional 3 h; and the cells were then subjected to the GSH assay. ** Significantly different (n = 3, *p*<0.01, Tukey, ANOVA). (B) Cell samples treated under indicated conditions were separated by 2D-PAGE and subsequently subjected to western blot analysis using the anti-DJ-1 antibody. The ratio of oxidized DJ-1 is shown under each condition (n = 2).

We also examined the effect of NAC on DJ-1 oxidation. Treatment with 2 mM NAC for 2 h resulted in a marked increase in cellular GSH (Fig. 8A). NAC-treated cells were washed and treated with 6-OHDA in fresh serum medium to examine the role of cellular GSH. 6-OHDA treatment significantly decreased cellular GSH levels (Fig. 8A), while a significant difference was not observed following control and 6-OHDA+NAC treatment. Thus, in the NAC-treated cells, 6-OHDA did not decrease cellular GSH to an amount lower than that of the control (Fig. 8A). For appropriate cellular GSH levels, NAC could prevent the oxidation of DJ-1 by 6-OHDA (Fig. 8B).

## Discussion

Several compounds such as H_2_O_2_ and 6-OHDA have been known to induce DJ-1 oxidation; however, the molecular mechanism underlying 6-OHDA-induced DJ-1 oxidation, especially the ROS responsible, has not been elucidated. In the present study, we clearly demonstrated that 6-OHDA-derived *p*-quinone plays a significant role in the DJ-1 oxidation and in decreasing cellular GSH.

It has been reported that H_2_O_2_ can induce DJ-1 oxidation; it is notable that a relatively high concentration of H_2_O_2_ is necessary for this oxidation as follows: 1 mM H_2_O_2_ for 60 min in HUVEC [14], 500 µM for 15 min in Jurkat cells [23], and 0.5—2.5 mM for 12 h in SH-SY5Y cells [10]. In the present study, treatment with 1 mM H_2_O_2_ for 3 h induced DJ-1 oxidation in SH-SY5Y cells; however, treatment with 600 µM H_2_O_2_ did not induce obvious oxidation (Fig. 4). A slight increase of oxDJ-1 in 600 µM H_2_O_2_-treated cells indicates the importance of *p*-quinone in 6-OHDA-induced DJ-1 oxidation in the light of stoichiometric considerations. The solution of 1 mM H_2_O_2_ was highly toxic and almost all cells died after treatment. On the other hand, 600 µM 6-OHDA in the presence of catalase was relatively mild and after treatment with this solution, the cell viability was more than 60%. In the case of BSO, the cell viability was small but significantly decreased to 70% of the control (data not shown). These observations suggest that DJ-1 oxidation is not correlated with cell viability. DJ-1 oxidation, induced by 6-OHDA with or without catalase and BSO, involved a decrease in GSH levels, while NAC treatment, in which the GSH levels in 6-OHDA-treated cells were the same as that of the control, completely suppressed DJ-1 oxidation. These observations point to the critical role of GSH levels in DJ-1 oxidation.

The multiple roles of GSH in the protection of cells against oxidative stress and other xenobiotic compounds have been well established [30]. It is therefore postulated that the dysfunction induced by a GSH decrease can be of different types. For example, the decrease in cellular GSH attenuates the enzyme activity of GSH peroxidases (GPxs) and GSH S-transferases (GSTs). GPxs remove several kinds of hydroperoxides such as H_2_O_2_ and lipid hydroperoxide [31], while GSTs catalyze the conjugation of GSH to xenobiotics and endogenous substrates with an electrophilic functional group, thereby decreasing their reactivity with cellular macromolecules [32]. It has also been reported that low levels of GSH lead to lipid peroxidation and the activation of enzymes such as lipoxygenases and phospholipases [33], [34]. In order to investigate the involvement of free radical-medicated lipid peroxidation in DJ-1 oxidation, the protective effects of vitamin E, one of the most potent free radical-scavenging antioxidants, was examined; however, the obvious protective effects of vitamin E on DJ-1 oxidation have not been observed (data not shown). The detailed molecular mechanisms by which oxidation of DJ-1 is caused due to the decrease in intracellular GSH levels are still under investigation.

Several studies have reported the role of DJ-1 in antioxidative defence systems; for example, the regulation of glutathione metabolism and the gene expression of 2 uncoupling proteins (UCP4 and UCP5) is considered as one of the promising molecular mechanisms of antioxidative action of DJ-1 [13], [35]. On the other hand, it has been shown that Cys-106 plays an important role in the antioxidative action of DJ-1 to prevent the cell death induced by ROS [10]. The present study showed that the decrease in cellular GSH level can induce DJ-1 oxidation (Fig. 7). On the basis of the findings obtained in this and previous studies, it is postulated that DJ-1 in antioxidative defence systems acts as a sensor for alteration in oxidative stress status, such as depletion of cellular GSH, to change the gene expression levels related to antioxidative defence systems. It has been reported that formation of Cys-SO_2_H at Cys-106 of DJ-1 is critical for its function. The Cys-SO_2_H form of DJ-1 is the active form, while the Cys-SO_3_H form of DJ-1 is the inactive form [36], [37]. Oxidation of the Cys residue to Cys-SO_2_H or Cys-SO_3_H results in an acidic pI shift of DJ-1. Although we did not distinguish between these 2 forms in the present study, the Cys-SO_2_H form of DJ-1 may be hypothesized to act as a sensor protein to upregulate the antioxidant system and combat ROS, while the overoxidized Cys-SO_3_H form of DJ-1 does not function in directing the cell to the cell death pathway.

It has also been postulated that DJ-1 plays a role in the antioxidative defence to remove H_2_O_2_ via the direct oxidation of Cys-106 in DJ-1; however, this reaction is not enzymatic [10], and it might be less functional compared with those induced by other antioxidative enzymes such as GSH peroxidases and catalase. Therefore, it might be possible that the biological meaning of DJ-1 oxidation is to detect the imbalance between oxidants and antioxidants rather than mere oxidative damage of DJ-1.

In conclusion, the present study clearly shows that electrophilic *p*-quinone formed from 6-OHDA induces DJ-1 oxidation via the depletion of cellular GSH. Previous studies have demonstrated that GSH levels in PD patients significantly decrease [8], while the involvement of oxidative modification of DJ-1 in the pathogenesis of PD has been suggested [16], [37]. Our results and the results of previous studies suggest that there is a relationship between the loss of cellular GSH and the DJ-1 oxidation in PD.

## Materials and Methods

### Materials

6-OHDA (purity: more than 97%), catalase, buthionine-SR-sulfoximine (BSO), and anti-beta actin (AC-15) were purchased from Sigma-Aldrich, St. Louis, MO; H_2_O_2_ and NAC, from Wako Pure Chemical Industries, Osaka, Japan; benzoquinone (BzQ) and tetramethylquinone (DuroQ) from Tokyo Chemical Industry, Tokyo, Japan; and GSH and 3-[4,5-dimethylthiazol-2-yl]-2,5-di-phenyltetrazolium bromide (MTT), from Nacalai Tesque, Kyoto, Japan. Dulbecco's modified Eagle medium: nutrient mixture F-12 Ham = 1∶1 (D-MEM/F-12) was purchased from Invitrogen, Carlsbad, CA, while fetal bovine serum (GPK0029) was purchased from Hyclone, Logan, UT. SH-SY5Y cells were obtained from the American Tissue Type Collection, Manassas, VA. Other chemicals were of the highest quality commercially available.

### Cell culture and determination of cell viability

Human neuroblastoma SH-SY5Y cells were routinely maintained in D-MEM/F-12 medium containing 10% heat-inactivated fetal bovine serum, and antibiotics (0.05 U/ml penicillin, 0.05 mg/ml streptomycin; Invitrogen) at 37°C in an atmosphere of air (95%) and CO_2_ (5%). To analyze DJ-1 oxidation induced by 6-OHDA, the SH-SY5Y cells were grown on plates, with a density of 2×10^5^ cells/ml. After the cells were attached (16–18 h), they were treated with different concentrations of 6-OHDA for a specific time. For the determination of cell viability, the MTT assay was conducted as described previously [38]. Briefly, the cells were incubated with 0.5 mg/ml MTT at 37°C for 4 h. Isopropyl alcohol containing 0.04 N HCl was added to the culture medium (3∶2, by volume), and they were mixed by using a pipette until the formazan was completely dissolved. The optical density of formazan was measured at 570 nm using an OPTImax plate reader (Molecular Devices).

In the case of primary cortical neuronal cells, the cells were isolated from the cerebral cortex of rat fetuses (Sprague-Dawley rats, day 17 of gestation; SLC, Sizuoka, Japan) as described previously [39]. More than 6 days after plating the cultured neurons with serum medium, the cells were treated with 6-OHDA. The use of animals was approved by the Animal Care and Use Committee of Doshisha University (approval No. 1008). All efforts were made to minimize animal suffering and to reduce the number of animals used.

### Protein extraction and protein assay

To obtain whole-cell extracts, the treated cells were corrected, washed with ice-cold PBS, and resuspended in lysis buffer (150 mM NaCl, 50 mM Tris-HCl, pH 7.4, 50 mM NaF, 5 mM ethylenediaminetetraacetic acid (EDTA), 0.5% Triton X-100 and 1 mM Na_3_VO_4_ with a protease inhibitor cocktail tablet (Nacalai Tesque)) at 4°C for 30 min. Nuclei and unlysed cellular debris were removed by centrifugation at 15,000× g for 5 min. The protein concentration was determined by using a bicinchoninic acid (BCA) protein assay kit (Pierce Biotechnology, Rockford, IL) with bovine serum albumin as the standard.

### 1D- and 2D- polyacrylamide gel electrophoresis (PAGE) for western blotting

Proteins in cell lysates were separated by 1D- and 2D-PAGE in a manner described previously [40]. In 1D-PAGE, a specific amount of protein (15 µg) from each sample was electrophoresed on 12.5% sodium dodecyl sulfate-polyacrylamide gel electrophoresis (SDS-PAGE). For the first dimension of 2D-PAGE, immobilized pH gradient (IPG) gel strips (pH 4–7; non-linear, 7 and 13 cm, GE Healthcare Bioscience, Uppsala, Sweden) were used. The samples (15 µg protein) were mixed with rehydration buffer (9 M urea, 5% CHAPS, 65 mM dithioerythritol (DTE), 0.5% ampholyte (pH 4–7)) and applied on a gel. The electrophoresis voltage was increased stepwise to 5000 V or 8000 V at a maximum current of 200 mA for 3–5 h. The second-dimensional separation was achieved by performing SDS-PAGE in the manner described above.

After the samples were separated by either 1D-PAGE or 2D-PAGE, they were transferred onto an Immobilon-P Transfer Membrane (Millipore, Bedford, MA). The membranes were blocked in 5% skim milk powder (Snow Brand Milk Products, Tokyo, Japan) dissolved in Tris-buffered saline (pH 7.4) containing 0.1% Tween 20 (TBS-T), incubated with goat anti-PARK7/DJ-1 polyclonal antibodies (Abcam, Cambridge, MA) or a mouse anti-oxDJ-1 monoclonal antibody (7411 clone) [16] at 4°C for 18 h, washed with TBS-T, incubated with horseradish peroxidase-conjugated secondary antibodies for at least 1 h, and washed with TBS-T. Immunoreactivity to these antibodies was visualized using Immobilon Western (Millipore) and LAS-4000 (Fujifilm, Tokyo, Japan). The relative densities of DJ-1 and oxDJ-1 were determined by Multi Gauge software (Fujifilm) and the ratio of the amount of oxDJ-1 to the total amount of DJ-1 was calculated from the following equation: the ratio = (intensity of oxidized DJ-1)/(intensity of DJ-1 + intensity of oxidized DJ-1). The ratio values shown are mean values obtained by considering a minimum of two independent experiments.

### *p*-Quinone and H_2_O_2_ generation from 6-OHDA

The amount of *p*-quinone generated in a cell-free system under conditions corresponding to cellular 6-OHDA treatments was measured spectrophotometrically using a previously described method [27]. Briefly, serum containing DMEM/F12 (phenol red-free) was thermostatically maintained at 37°C during the experiment. The experiment was initiated by adding of 6-OHDA to final concentrations of 0–800 µM. Maximum absorption of *p*-quinone (490 nm) was measured every 10 s. This reaction was completed within 10 min. The concentration of *p*-quinone generated was calculated using a molecular extinction coefficient of 1892 M^−1^ cm^−1^ at 490 nm for *p*-quinone [27].

H_2_O_2_ concentrations in a cell-free system under conditions corresponding to cellular 6-OHDA treatments were determined using FOX assay in a manner described previously [41]. Briefly, 600 µM 6-OHDA was added to serum containing DMEM/F12 (phenol red-free) with or without 50 U/ml catalase, and the medium was incubated for 15 min at 37°C. At specific time points, reaction mixtures were added to FOX reagent consisting of 100 µM xylenol orange, 250 µM ammonium ferrous sulfate, 100 mM sorbitol, and 25 mM H_2_SO_4_. Absorption was measured at 560 nm, and the concentration of H_2_O_2_ was calculated using a standard curve prepared with H_2_O_2_ in the same culture media.

### Determination of cellular GSH content

The intracellular GSH content was enzymatically determined by using 5,5′-dithiobis-(2-nitrobenzoic acid), according to a method described previously [42]. The GSH content was calculated by using reduced GSH (Nacalai Tesque) as the standard. The protein concentration was determined by using a BCA protein assay kit (Pierce Biotechnology, Rockford, IL) with bovine serum albumin as the standard.

### Statistical analysis

The statistical significance of differences between determinations was calculated by analysis of variance (ANOVA) using Tukey test for multiple comparisons. Data are reported as mean values ± SD. Values of *p*<0.05 were considered significant.

## Supporting Information

Figure S1 Effects of SOD and L-NMMA against DJ-1 oxidation induced by 6-OHDA. (A) Cell samples treated with 600 µM 6-OHDA in the presence or absence of 10 µg/ml SOD for 3 h were separated by 2D-PAGE and subsequently subjected to western blot analysis using the anti-DJ-1 antibody. The ratio of oxidized DJ-1 is shown under each condition (n = 2). (B) Cells were pretreated with or without 500 µM L-NMMA for 3 h, and this was followed by treatment with 600 µM 6-OHDA for an additional 3 h; and the cell samples were separated by 2D-PAGE and subsequently subjected to western blot analysis using the anti-DJ-1 antibody. The ratio of oxidized DJ-1 is shown under each condition (n = 2).
(EPS)
